# In Response

**DOI:** 10.1213/ANE.0000000000008137

**Published:** 2026-08-26

**Authors:** Anne M. Beukers, Markus W. Hollmann, Susanne Eberl, Henning Hermanns

**Affiliations:** Department of Anesthesiology, Amsterdam University Medical Center, Amsterdam, the Netherlands; Department of Anesthesiology, Amsterdam University Medical Center, Vrije Universiteit, Amsterdam, the Netherlands, a.beukers@amsterdamumc.nl; Department of Anesthesiology, Amsterdam University Medical Center, Amsterdam, the Netherlands; Department of Anesthesiology, Amsterdam University Medical Center, University of Amsterdam, Amsterdam, the Netherlands

## To the Editor

We thank Dr Grocott^1^ for highlighting the importance of precise terminology. We fully agree that the term “decarboxylation” is inaccurate in the context of cardiopulmonary bypass (CPB). As correctly noted, decarboxylation refers to a specific biochemical reaction, whereas CPB’s relevant function is the removal of carbon dioxide, reflecting its role in extracorporeal ventilation. Clarifying this distinction ensures greater accuracy in describing CPB physiology.^1^

We also agree that reductions in minimum alveolar concentration (MAC) are a critical consideration when selecting appropriate anesthetic target concentrations. In routine surgical settings where CPB is not used, volatile anesthetic administration is typically guided by end-tidal concentrations, which closely approximate cerebral partial pressures and thus serve as an indicator for anesthetic depth.^2^ However, the introduction of CPB challenges this conventional paradigm in several important ways.

First, while we emphasized that CPB-related factors, such as hypothermia and hemodilution, may reduce sevoflurane plasma concentrations,^3^ these factors may simultaneously increase anesthetic potency. However, the relative contribution and causal interplay of factors known to reduce MAC remain incompletely understood.

As discussed in our review,^4^ the initial decrease in plasma concentration of volatile anesthetics is followed by a gradual increase during CPB due to CPB-related factors, including hypothermia, and anesthetic uptake by the oxygenator.^3,5^ In addition, patient-related factors may further influence anesthetic behavior during CPB. These include increased tissue capacity for volatile anesthetics,^3^ and subtle increased cerebral injury, inflammatory responses, and alterations in blood–brain barrier function, as emphasized by Dr Grocott.^1^ Furthermore, interpatient variability in blood/gas solubility may influence volatile anesthetic kinetics. For example, patients with coronary artery disease often show elevated serum triglyceride levels, which can increase the blood/gas solubility of isoflurane and slow equilibration between inspired and alveolar concentrations compared with healthy individuals.^6^ Conversely, although children with cyanotic congenital heart disease have higher hematocrit levels than those with acyanotic conditions, similar blood/gas solubilities have been reported between these groups.^6^ Taken together, these findings suggest that the pharmacokinetics and pharmacodynamics of volatile anesthetics during CPB are shaped by a complex interplay of both CPB-related and patient-specific factors, limiting the predictability of volatile anesthetic behavior and highlighting the need for more individualized approaches to anesthetic management. The uncertainties highlighted in our review^4^ and further underscored by Dr. Grocott’s commentary^1^ collectively reinforce the need for dedicated prospective studies that systematically address the pharmacokinetic and pharmacodynamic complexities of volatile anesthetic management during CPB.


**Anne M. Beukers, MD, PhD** *Department of Anesthesiology* *Amsterdam University Medical Center* *Amsterdam, the Netherlands* *Department of Anesthesiology* *Amsterdam University Medical Center* *Vrije Universiteit* *Amsterdam, the Netherlands* *a.beukers@amsterdamumc.nl*
**Markus W. Hollmann, MD, PhD** **Susanne Eberl, MD, PhD** **Henning Hermanns, MD, PhD** *Department of Anesthesiology* *Amsterdam University Medical Center* *Amsterdam, the Netherlands* *Department of Anesthesiology* *Amsterdam University Medical Center* *University of Amsterdam* *Amsterdam, the Netherlands*


## References

[1] Grocott HP. Cardiopulmonary bypass effects on minimum alveolar concentration and other bypass terminology. Anesth Analg. 2026;143:e46–e47. 10.1213/ANE.0000000000008136 42202967

[2] Hendrickx JFA De Wolf AM. End-tidal anesthetic concentration: monitoring, interpretation, and clinical application. Anesthesiology. 2022;136:985–996. 35483048 10.1097/ALN.0000000000004218

[3] Meroni R Gianni S Guarnieri M. Feasibility of anesthesia maintenance with sevoflurane during cardiopulmonary bypass: a pilot pharmacokinetics study. J Cardiothorac Vasc Anesth. 2017;31:1210–1217. 28283250 10.1053/j.jvca.2016.12.018

[4] Beukers A Breel J van den Brom C. Pharmacokinetics and pharmacodynamics of analgesic and anesthetic drugs in patients during cardiac surgery with cardiopulmonary bypass: a narrative review. Anesth Analg. 2026;142:5–14. 40378083 10.1213/ANE.0000000000007564 PMC12677336

[5] Nitzschke R Wilgusch J Kersten JF. Changes in sevoflurane plasma concentration with delivery through the oxygenator during on-pump cardiac surgery. Br J Anaesth. 2013;110:957–965. 23462192 10.1093/bja/aet018

[6] Hu P Zhou JX Liu J. Blood solubilities of volatile anesthetics in cardiac patients. J Cardiothorac Vasc Anesth. 2001;15:560–562. 11687994 10.1053/jcan.2001.26531

